# Semi-Automated, Occupationally Safe Immunofluorescence Microtip Sensor for Rapid Detection of *Mycobacterium* Cells in Sputum

**DOI:** 10.1371/journal.pone.0086018

**Published:** 2014-01-22

**Authors:** Shinnosuke Inoue, Annie L. Becker, Jong-Hoon Kim, Zhiquan Shu, Scott D. Soelberg, Kris M. Weigel, Morgan Hiraiwa, Andrew Cairns, Hyun-Boo Lee, Clement E. Furlong, Kieseok Oh, Kyong-Hoon Lee, Dayong Gao, Jae-Hyun Chung, Gerard A. Cangelosi

**Affiliations:** 1 Department of Mechanical Engineering, University of Washington, Seattle, Washington, United States of America; 2 Department of Environmental and Occupational Health Sciences, University of Washington, Seattle, Washington, United States of America; 3 Departments of Medicine-Division of Medical Genetics and Genome Sciences, University of Washington, Seattle, Washington, United States of America; 4 NanoFacture, Inc., Bellevue, Washington, United States of America; McGill University, Canada

## Abstract

An occupationally safe (biosafe) sputum liquefaction protocol was developed for use with a semi-automated antibody-based microtip immunofluorescence sensor. The protocol effectively liquefied sputum and inactivated microorganisms including *Mycobacterium tuberculosis*, while preserving the antibody-binding activity of *Mycobacterium* cell surface antigens. Sputum was treated with a synergistic chemical-thermal protocol that included moderate concentrations of NaOH and detergent at 60°C for 5 to 10 min. Samples spiked with *M. tuberculosis* complex cells showed approximately 10^6^-fold inactivation of the pathogen after treatment. Antibody binding was retained post-treatment, as determined by analysis with a microtip immunosensor. The sensor correctly distinguished between *Mycobacterium* species and other cell types naturally present in biosafe-treated sputum, with a detection limit of 100 CFU/mL for *M. tuberculosis*, in a 30-minute sample-to-result process. The microtip device was also semi-automated and shown to be compatible with low-cost, LED-powered fluorescence microscopy. The device and biosafe sputum liquefaction method opens the door to rapid detection of tuberculosis in settings with limited laboratory infrastructure.

## Introduction

*Mycobacterium tuberculosis*, the bacterium responsible for tuberculosis (TB), remains a significant global public health threat, claiming over a million lives each year [1]–[3]. Rapid diagnosis is important not only for patient care but also to prevent disease transmission. In addition to common aerosol transmission of the pathogen in communities, occupational infection of healthcare workers and laboratory personnel can occur through multiple routes. Due to their exposures, these workers are at increased risk of both TB infection and disease [4], [5].

The standard protocols for detecting *M. tuberculosis* in sputum samples are smear microscopy and bacterial culture. Sputum smear microscopy is the major diagnostic tool used in many resource-limited settings [6], [7]. The method lacks sensitivity but is relatively rapid, simple, and inexpensive in areas with high prevalence of TB [8]. Bacterial culture is more sensitive than microscopy but may take several weeks to yield results [9]. Laboratory infrastructure is needed for either microscopy or culture, and the latter requires biosafety level 3 (BSL3) containment to minimize the risk of occupational exposure to the pathogen.

With the introduction of the GeneXpert (Cepheid, Sunnyvale, CA, USA), rapid nucleic acid amplification tests are becoming more common in clinical laboratories. The automated, cartridge-based polymerase chain reaction (PCR) assay can be operated outside of BSL3 labs and can diagnose smear-positive TB in two hours with 98% reliability [10]. Although far more rapid than culture-based methods, a two-hour time to result may be too long for some patient visits. For example, an implementation study reported revisions to procedures and interventions to address the problem of patients leaving the clinic or becoming untraceable within the clinic prior to completion of the 2-hour GeneXpert protocol [11]. Moreover, the GeneXpert instrument is expensive to install and operate [3], [6]. Thus, there remains a need for an inexpensive and rapid assay to detect TB.

Newer technologies enable the rapid detection of pathogen antigens (either whole-cell or molecular) without requiring nucleic acid amplification and with sensitivity approaching that of PCR [12], [13]. For example, we reported a microtip immunofluorescence sensor capable of detecting 200 CFU/mL of *M. tuberculosis* complex cells in spiked sputum [14]. Using microscale silicon tips, *M. tuberculosis* cells were concentrated and captured from sputum by electrohydrodynamic effects. The captured cells were detected by low-magnification (20X objective) fluorescence microscopy. Specificity was conferred by a polyclonal antibody that was immobilized on the microtip surfaces. The entire process required 25 minutes to complete from raw sample to numerical result, making it significantly faster than any nucleic acid amplification test.

A limitation of antigen detection is the requirement for relatively gentle sample handling. Antigen structure must be preserved to enable detection by antibodies or other molecular probes. This requirement is problematic when working with infectious samples such as sputum from TB patients. For example, sputum samples in our previous report were treated with N-Acetyl-L-Cysteine (NALC) and sodium dodecyl sulfate (SDS) [14]. This process liquefied the viscous sputum samples while preserving antigen integrity, however it did not disinfect the samples. As a result, BSL2 or BSL3 laboratory infrastructure would be required for safe operation when applied to samples from TB patients. Similar limitations are likely to apply to other antigen detection platforms.

In this paper, we addressed the challenge of inactivating *Mycobacterium* cells and other pathogenic organisms in sputum, while maintaining mycobacterial cell integrity for concentration and immunodetection using the microtip assay. *Mycobacterium* cells are robust and difficult to inactivate without harming antigenic structures. In addition, residual microbicides could “poison” antigen-antibody interactions. To address this challenge, we investigated a synergistic approach, in which moderate heating was used to transiently increase the susceptibility of *Mycobacterium* cells to moderate disinfectant treatment. This strategy was based in part on reports that the waxy, impermeable mycobacterial cell envelope undergoes a reversible phase shift at around 60°C, resulting in a semi-fluid structure [15]. We hypothesized that the altered structure would render the cells more susceptible to damage by mild chemical challenges such as NaOH and detergents. A process was thereby developed that enables the safe molecular detection of *Mycobacterium* cells in settings that lack BSL3 or even BSL2 containment.

This paper also describes improvements to our microtip platform that renders the process more user-friendly. The improved device is semi-automated to reduce labor and to improve the reproducibility of washing and immunofluorescence detection. To characterize the performance of the improved device and protocols, the device was tested for detection of *M. tuberculosis* complex (strains H37Ra and BCG Russia), non-tuberculous *Mycobacterium* (NTM) species (*M. avium* strain 104 [16] *and M. smegmatis* mc^2^155), and *Staphylococcus epidermidis* spiked into sputum samples. To render the assay more affordable, a low-cost, battery operated fluorescence microscopy system was validated as a replacement to a previously-used high-resolution system. The biosafe, semi-automated immunofluorescence microtip sensor completed detection of *Mycobacterium* cells in 30 minutes with a detection limit of 100 CFU/mL in sputum.

## Materials and Methods

### Biosafe sputum liquefaction protocol

**Figure 1:**
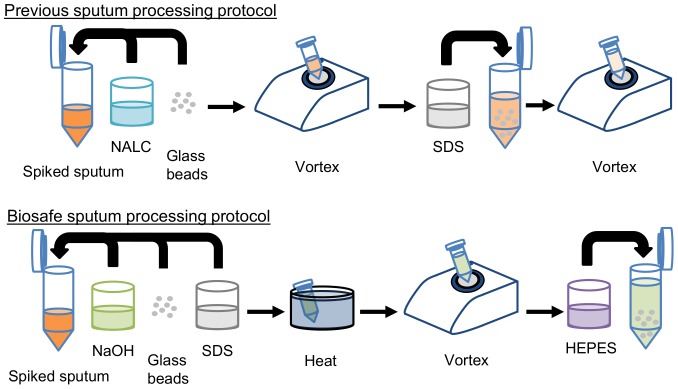
Comparison of the previous protocol (top) and biosafe sputum processing protocol (bottom).

Human sputum was purchased from Bioreclamation, LLC (Westbury, NY) and stored in 300 µL aliquots in cryogenic tubes at −80°C until use. Prior to running experiments, frozen samples were thawed at room temperature and spiked with cultured bacteria where indicated. To run the biosafe protocol, 600 µL of spiked sputum (300 µL of sputum with 300 µL of bacterial cell suspension suspended in PBS) was supplemented with 300 µL NaOH solution (0.4 M), 300 µL sodium dodecyl sulfate solution (4% SDS), and 15 glass beads with diameter of 3 mm. The suspension was vortexed briefly. Tubes were incubated in a 60°C water bath for 10 minutes, then vortexed at 1400 rpm for 5 minutes. Subsequently, 1 mL out of the 1.2 mL suspension from each tube was transferred to an empty tube and 250 µL HEPES buffer (1 M) was added to each tube to neutralize the NaOH. The current and previous sputum processing protocols are shown in Figure 1.

### Bacteriological culture

Bacteria used to spike samples were cultivated in a shaker incubator at 37°C until cultures were saturated. *Mycobacterium* strains were cultured in Difco Middlebrook 7H9 Broth (BD Diagnostics, Sparks, MD) supplemented with 10% (v/v) ADC enrichment and 0.05% Tween 20. Other organisms were grown in trypticase soy broth.

### Microbiological analysis of biosafe protocol-treated samples

Cultured *M. tuberculosis* complex cells were suspended either in phosphate buffered saline (PBS) or in human sputum mixtures as described above, at estimated densities of approximately 1×10^7^ CFU/mL sputum [calculated based on optical density (A_600_) measurement of cultures]. After the various treatments described in this report, 0.1 mL samples of the suspensions were plated in triplicate onto Difco Middlebrook 7H10 agar with 10% (v/v) OADC enrichment (BD Diagnostics, Sparks, MD). Plates were incubated at 37°C for up to 35 days. Untreated sputum samples rapidly overgrew plates due to the natural flora of sputum. Therefore, no-treatment controls consisted of untreated suspensions of *M. tuberculosis* complex cells in PBS. This strategy enabled us to quantify viable *M. tuberculosis* complex cells in untreated controls.

### Microtip assay

**Figure 2:**
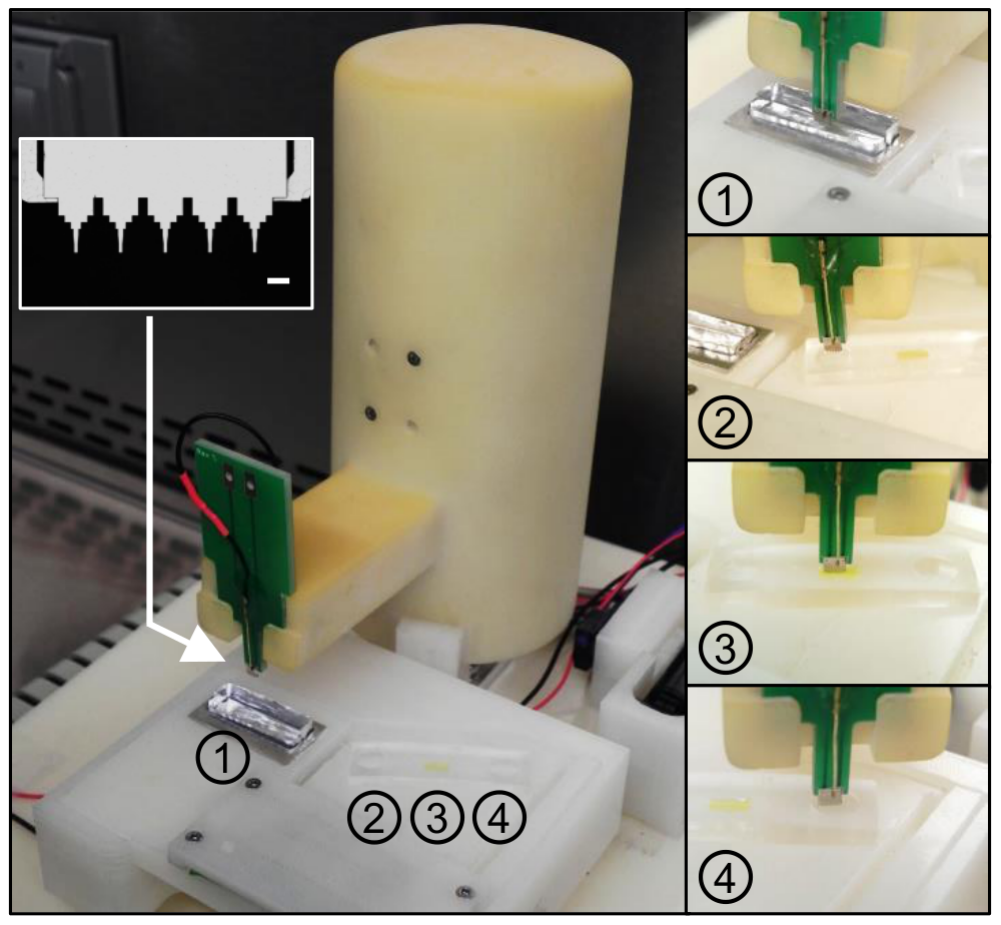
Semi-automated microtip system for concentration and detection of *M. tuberculosis*. The microtip used in the device is shown with scale bar of 250 µm. Components are as follows: (1) Sample well in which cells are concentrated and captured onto the microtip, (2) initial rinsing well with 1% SDS solution, (3) labeling well in which cells captured on microtip are bound to fluorescent antibody, (4) final rinsing well with DI water.

The semi-automated, immunofluorescence microtip assay, a new design based on our previous device [14], is shown in Figure 2. Further information on the device operation and mechanism is described in the supplementary text and Figure S1, both of which are in File S1. A microtip decorated with antibodies to specifically capture target bacteria in sputum is installed on a coupon and automatically dipped and withdrawn from the sample well (well number 1 in Figure 2). The tip is then automatically pivoted to wells with rinsing solutions and reporter antibody, numbered from 2 through 4 in Figure 2.

In an indication of the reproducibility of these methods, the experiments reported here were conducted over a period of 4 months and fresh bacterial cell cultures were used each week. Fresh batches of functionalization reagents and microtips were used each month. Sputum samples were purchased in several batches. In addition, three different research personnel operated the semi-automated microtip assay with comparable results. In this fashion, variability related to sample composition, target cell physiology, and human errors were evaluated.

## Results

### Inactivation of M. tuberculosis in sputum

We hypothesized that the reversible phase shift that occurs in mycobacterial cell walls at around 60°C [15] would render the cells more susceptible to damage by mild chemical challenges such as 0.1 M NaOH and 1% SDS. To test this hypothesis, we developed and evaluated the biosafe protocol described in Methods and shown in the bottom row of Figure 1. The previous sputum processing protocol [14] is shown in the top row of Figure 1.

Experiments were performed to evaluate the effects of the new protocol on the viability of *M. tuberculosis* complex cells. Initial experiments were conducted on *M. bovis* BCG cells suspended in PBS. Suspensions were exposed to varying temperatures (room temperature to 80°C), for varying periods of time (2 to 15 minutes), and with varying concentrations of NaOH (0 to 1 M). At room temperature, protocols utilizing NaOH at concentrations up to 1 M did not significantly reduce the viability of BCG in PBS. When cells were treated with elevated temperatures (50°C, 55°C, and 60°C) in PBS in the absence of chemical challenges, only partial loss of viability was observed (lawns of BCG colonies on plates were visibly thinned but still too numerous to count). In contrast, the complete biosafe protocol, which involved treatment with 0.1 M NaOH and 1% SDS (final concentrations) at 60°C for ≥5 minutes, reduced the viability of BCG approximately 10^6^-fold (Table 1). Shorter incubation times (2 min) or lower temperatures (room temperature or 50°C) were less effective. In two additional experiments, 5 to 10 min of treatment at 60°C with 0.1 M NaOH and 1% SDS always resulted in ≥10^6^-fold loss of viability of BCG. To maximize occupational safety a 10-min protocol was used in subsequent experiments.

**Table 1:** Effects of temperature and treatment time on inactivation of BCG cells in PBS.

Treatment time	Colony forming units (CFU)^1^		
	Room temperature	50°C	60°C
**0 min (untreated)**	TNTC^2^	-	-
**2 min**	TNTC	TNTC	234
**5 min**	TNTC	TNTC	3
**10 min**	TNTC	TNTC	3
**15 min**	TNTC	TNTC	1

1 BCG cells were suspended in PBS at concentrations of approximately 1×10^7^ CFU/mL. After treatment of 0.1 M NaOH and 1% SDS, 0.1 mL aliquots were plated on triplicate Middlebrook 7H10 plates with OADC enrichment. Numbers shown are total counts for the three plates after 35 days of incubation at 37°C.

2 TNTC, too numerous to count.

To test the protocol's effect on microorganisms present in unspiked sputum, we plated both untreated and treated sputum on Middlebrook 7H10 media. The untreated plates were found to be overgrown with a variety of both bacteria and fungi, while the biosafe protocol inactivated all culturable (on Middlebrook medium) microorganisms present in the sputum samples.

Subsequent experiments were conducted on cultured cells of *M. tuberculosis* strains H37Ra and H37Rv spiked into human sputum. The complete 10-min biosafe protocol inactivated both *M. tuberculosis* strains in sputum by ≥10^6^-fold. Table 2 shows the results of six experiments conducted on separate sputum samples spiked with *M. tuberculosis* H37Rv. Samples were subjected to the 10-min biosafe protocol with and without bead beating. The protocol consistently resulted in ≥10^6^-fold inactivation of H37Rv regardless of bead beating. Although bead beating was not required for pathogen inactivation, we believe it facilitated consistent microtip enrichment by reducing sputum viscosity. Subsequent experiments used the complete protocol with bead beating.

**Table 2:** Inactivation of *M. tuberculosis* H37Rv in sputum by using the biosafe protocol.

Treatment	Colony forming units (CFU)^1^					
	Sample 1	Sample 2	Sample 3	Sample 4	Sample 5	Sample 6
**No treatment** 2	TNTC^3^	TNTC	TNTC	TNTC	TNTC	TNTC
**With bead beating**	0	0	0	0	0	0
**Without bead beating**	0	0	0	0	0	0

1 H37Rv cells were suspended in sputum samples at concentrations of approximately 1×10^7^ CFU/mL. After treatment, 0.1 mL aliquots were plated on triplicate Middlebrook 7H10 plates with OADC supplement. Numbers shown are total counts for the three plates after 33 days of incubation at 37°C.

2 Because untreated sputum samples rapidly overgrew plates due to the natural flora of sputum, no-treatment controls consisted of *M. tuberculosis* cells suspended in PBS.

3 TNTC, too numerous to count.

### Microtip test

The previous microtip device [14] was remodeled to automate the steps from pathogen cell capture to fluorescence measurement. The modifications described in Methods reduced manual labor and the potential for operator errors. The semi-automated device was evaluated in combination with the biosafe sample processing protocol. The current study also extended previous evaluations (6) by assessing the specificity of the polyclonal antibody for *Mycobacterium* species other than *M. tuberculosis* complex.

**Figure 3:**
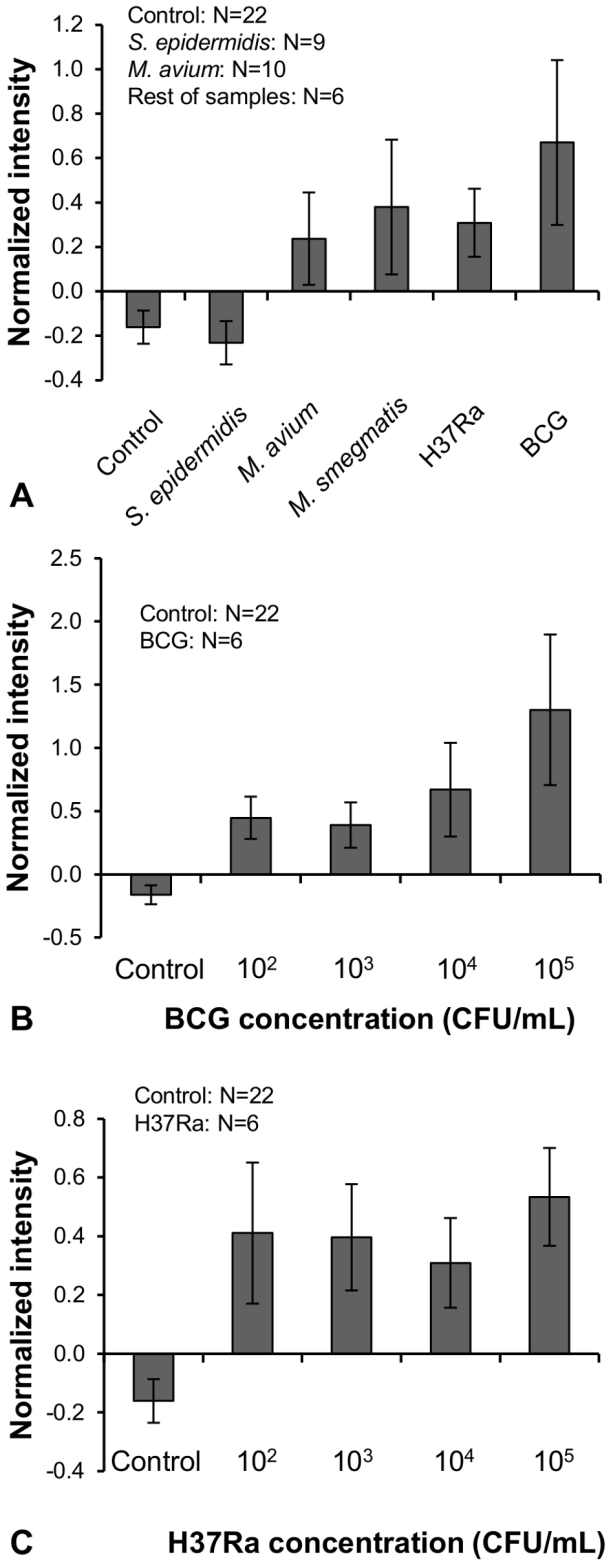
Normalized fluorescence intensity results from the microtip assay. (A) Comparison of different species of *Mycobacterium* and *S. epidermidis* at 10^4^ CFU/mL. (B) Microtip detection of treated sputum samples spiked with BCG at densities ranging from 10^2^ to 10^5^ CFU/mL. (C) Microtip detection of treated sputum samples spiked with H37Ra at densities ranging from 10^2^ to 10^5^ CFU/mL.

The investigation tested a total of 95 separate samples in spiked sputum, including 22 control samples spiked with PBS, 9 with *S. epidermidis* (10^4^ CFU/mL), 6 with *M. smegmatis* (10^4^ CFU/mL), 10 with *M. avium* (10^4^ CFU/mL), and 6 samples for each of four concentrations of BCG and *M. tuberculosis* H37Ra ranging from 10^2^ to 10^5^ CFU/mL (Fig. 3). Bacterial densities were estimated by 10-fold dilutions from 10^7^ CFU/mL. After spiking into sputum, the samples were processed by the biosafe protocol. After microtip analysis of the processed samples, fluorescent intensity values were normalized by the equation [Normalized intensity  =  (fluorescence intensity-19.2)/19.2]. The value of 19.2 was the average intensity of negative control assays + standard deviation. The error bars represent 95% CI.

Sputum spiked with *Mycobacterium* and processed by the biosafe protocol generated consistently positive signals (Figure 3a). A *t*-test with 95% CI was used for significance. Signals generated with *M. avium*, *M. smegmatis*, H37Ra, and BCG differed significantly from negative (non-spiked) control samples (*p* = 0.004, 0.01, <0.001, and 0.005, respectively). Samples spiked with *S. epidermidis* were statistically indistinguishable from negative controls.

*S. epidermidis* was chosen as a specificity control because of its common occurrence in all types of human tissue samples. In addition, the sputum itself was likely to have contained numerous human and microbial cells. Therefore, the specificity of the assay for *Mycobacterium* cells, when conducted on biosafe-treated samples, was evident. However, the assay cross-reacted with the NTM species *M. avium* and *M. smegmatis*. This cross-reactivity was likely to be a feature of the polyclonal antibody, not a result of the biosafe protocol, because untreated NTM cells were also observed to cross-react with this antibody (data not shown).

**Figure 4:**
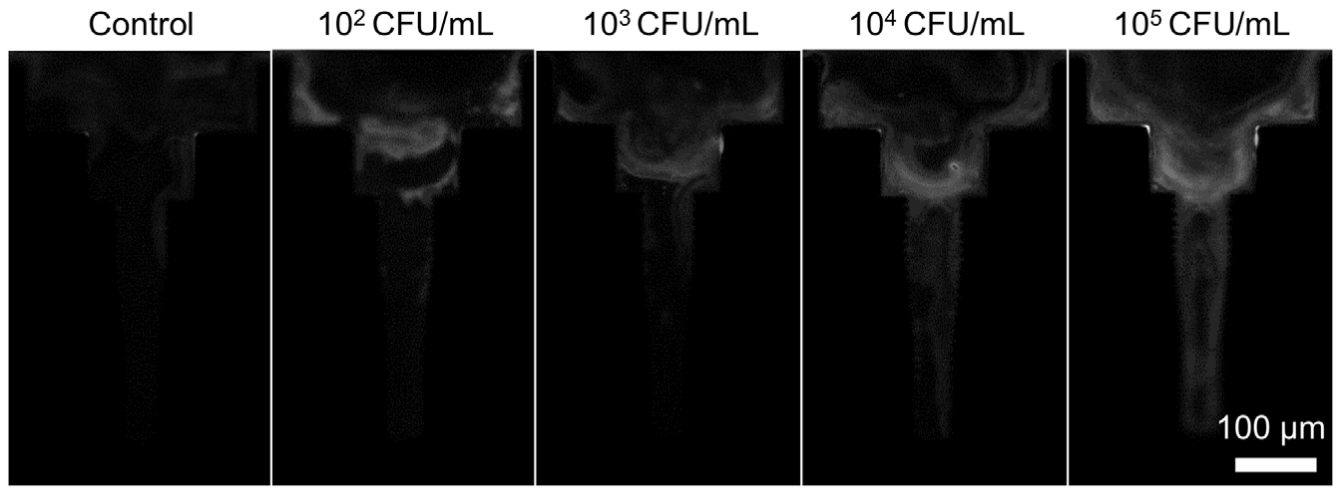
Raw fluorescent images (20X objective) of microtips after capture from spiked sputum samples with BCG at densities ranging from 0 (control) to 10^5^ CFU/mL.

The limit of detection of the semi-automated microtip immunofluorescence assay, when conducted on biosafe-treated samples, was determined by testing estimated concentrations of BCG and MTB H37Ra ranging from 10^2^ to 10^5^ CFU/mL (Fig. 3B, 3C). BCG at or above 10^2^ CFU/mL differed significantly from negative controls (*p*≤0.004 at all concentrations). The same was observed for H37Ra at or above 10^2^ CFU/mL (p≤0.003 at all concentrations). Thus, the detection limit achieved for both BCG and H37Ra was 100 CFU/mL, similar to the limit reported for samples treated by the previous NALC-SDS method (200 CFU/mL) [14]. As in the previous report, the dose-response curve saturated at low cell concentrations and was not strongly linear. Raw images of microtips after capturing serial dilutions of BCG concentrations are shown in Figure 4.

### Compatibility with low-cost fluorescence microscopy

**Figure 5:**
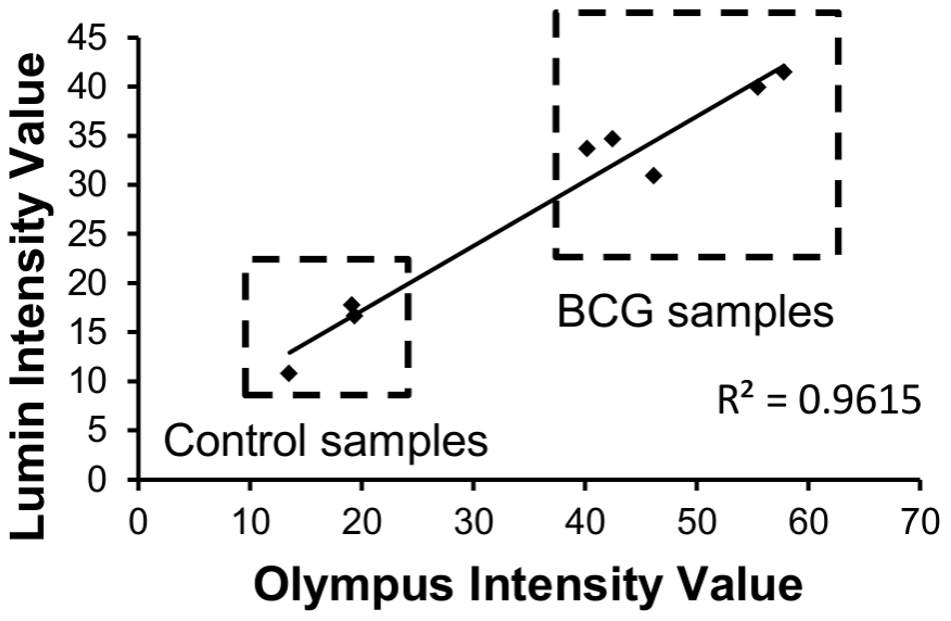
Preliminary comparison between Olympus and Lumin raw intensity values obtained from microtip measurement. The microtips were evaluated in treated sputum samples spiked with either PBS alone (control) or PBS containing BCG cells at 10^4^ CFU/mL.

In order to assess the compatibility of the microtip method with low-cost portable fluorescent microscopes, 8 sputum samples were assayed using the LED-based, battery-powered Lumin™ microscope (LW Scientific, Lawrenceville, GA, USA), along with the more expensive Olympus BX-41. Among the 8 samples, 3 were negative controls and the other 5 were spiked with BCG (10^4^ CFU/mL). The Lumin instrument yielded intensity values that were slightly lower than those of the Olympus, however both microscopes distinguished spiked from control samples with similar efficiency (Figure 5).

## Discussion

In the present study, heating to 60°C for 5 to 10 minutes in the presence of 1 M NaOH and 1% SDS was sufficient to kill the cells by a factor of approximately 10^6^-fold. Because heat transfer can vary depending on equipment and labware, a 10 minute heat treatment was used to confer a margin of error. We hypothesize that our method exploits the reversible phase shift of the mycobacterial cell wall that occurs at around 60°C [15]. Heating to the transition temperature may reduce the cell's structural resistance or resilience in the face of chemical challenges.

Previous observations found that high temperatures were needed to inactivate *M. tuberculosis* in the absence of chemical challenges. Zwadyk and colleagues [17] reported 50% inactivation after 95°C in a dry heat block for 20 minutes. Bemer-Melchior and Drugeon reported 20% inactivation after 20 minutes at 80°C [18]. However, Doig and colleagues reported that heating to 80°C for 20 minutes fully submerged in a water bath was sufficient to kill all the cells [19]. In all of these reports, temperatures ≥80°C were required to inactivate significant percentages of *M. tuberculosis* cells. Our results demonstrate that simultaneous exposure to chemical challenges (0.1 M NaOH and 1% SDS) can lower the temperature requirement to 60°C, a level that may in the future enable the use of chemical- or battery-powered heat supplies to render patient samples safe for analysis [20].

The liquefaction and disinfection protocol described here differs from the standard NALC-NaOH sputum processing protocol [21], [22]. NALC-NaOH maintains the viability of at least some *Mycobacterium* cells while inactivating rapidly-growing microorganisms in sputum. Therefore, it does not render a sample safe for handling outside of BSL2 or BSL3 settings.

Integrity of the surface antigens were a concern after sputum treatment with chemicals and heat. Loss of integrity of antigens could preclude the use of antibody-based detection protocols such as the microtip sensor. To test this possibility, we initially attempted to use an enzyme-linked immune-sorbent assay (ELISA) to characterize antigen integrity after the biosafe treatment. However, the presence of sputum resulted in high background signals regardless of treatment (not shown). Therefore, antigen activity was evaluated by using the immunofluorescent microtip assay. Samples treated by the biosafe method exhibited detection results that were essentially identical to previous results generated by using the NALC method [14]. The assay detected *M. tuberculosis* complex cells with a limit of detection of approximately 100 CFU/mL, in less than 30 minutes (sample to result), with no cross-reactivity to an unrelated bacterium (*S. epidermidis*) or to other microorganisms present in multiple sputum samples. It cross-reacted with other *Mycobacterium* species, but this appears to be an innate property of the polyclonal antibody, which was raised in chickens against whole acetone-fixed BCG cells [14].

The current configuration of the microtip assay requires fluorescence detection, which can be expensive depending on the instrument used. To determine if the method can utilize inexpensive fluorescence microscopy equipment, we compared fluorescent intensity values obtained by using the battery-powered LED of Lumin fluorescence microscope to those obtained by using the more elaborate and expensive Olympus BX-41 unit. The development of light emitting diodes (LED) with fluorescent capabilities and low power requirements has led to the recent introduction of robust, inexpensive, battery-powered fluorescent microscopes such as the Lumin instrument [23], [24]. Given that microtip visualization requires modest magnification (20X objective) and uses a well-defined focal plane, we hypothesized that a low-cost instrument could function as well as a high-end one. Although the Lumin's overall raw fluorescence intensity values were lower than those of the Olympus, the results were comparable.

The microtip assay and sputum processing protocol described here are rapid and sensitive, and can potentially be operated with low power consumption. However, several limitations remain to be addressed. Further development is needed to decrease manual steps, especially prior to sputum disinfection. The evaluated sputum samples were spiked with cultured *Mycobacterium* cells and did not come from patients with active tuberculosis. Because the current antibody cross reacts with NTM species, the specificity of the assay is not markedly improved over sputum smear microscopy (although analytical sensitivity is substantially improved). A monoclonal antibody specific to *M. tuberculosis* complex will be needed to increase the specificity. Lastly, we have not conducted detailed studies on the reproducibility of the biosafe protocol and microtip assay. These factors will be addressed with further development. The current study lays the foundation for such development by demonstrating the physical, chemical, and biological feasibility of the assay design.

In summary, an occupationally safe (biosafe) sputum processing protocol was developed for rapid diagnosis of *Mycobacterium* using an updated, semi-automated immunofluorescence-based microtip sensor. A synergistic strategy combining moderate chemical and thermal treatments for 10 minutes inactivated *M. tuberculosis* and other potential pathogens in sputum, while maintaining the integrity of mycobacterial surface antigens. When applied to biosafe-treated samples, the semi-automated microtip immunofluorescence sensor exhibited the predicted specificity and a detection limit of 100 CFU/mL. The detection time from untreated sputum sample to the final result was 30 minutes with analytical sensitivity comparable to commercial PCR [25]. In addition, the device was semi-automated and the protocol was shown to work well with low-cost, LED-powered microscopy. The new device and the biosafe protocol may prove useful in the future for TB diagnosis in settings with limited laboratory infrastructure.

## Supporting Information

File S1 (PDF)
