# Supplementary material for: Semi-Automated, Occupationally Safe Immunofluorescence Microtip Sensor for Rapid Detection of Mycobacterium Cells in Sputum
Source: PLoS One. 2014 Jan 22;9(1):e86018. doi: 10.1371/journal.pone.0086018 (PMC3899086; doi:10.1371/journal.pone.0086018)
Supplement: File S1 — (PDF) [file pone.0086018.s001.pdf]

Supplemented materials:

Microtip assay device operation and mechanism:

The microtip immunosensor device (Fig. 2) was designed for rapid identification of *M. tuberculosis* in sputum samples. The sensor is a microtip with immobilized polyclonal IgY antibodies on its surface. The antibodies were raised against whole acetone-treated *M. tuberculosis* complex cells. The device has a disposable aluminum well to hold the treated sputum. In addition, a PDMS well holds the rinsing solutions of deionized water (DI water) and SDS solutions (1% in DI water) as well as the fluorescent antibodies for labeling. Housed in the base under the wells are a vibration motor used during the capture process, and two linear motors to precisely control the position of the microtip sensor.

The operation begins with the functionalized microtip being immersed into aluminum well containing 1 mL of treated sputum (Fig. 2-1). Cells are rapidly concentrated to the microtip by a circulating flow produced from mechanical vibration, working in concert with electrokinetics from an applied AC field (Fig. S1). The vibration motor placed under the aluminum well generates a convective flow to bring cells spaced throughout the volume of the solution toward the microtip. Cells near the vicinity of the microtip are polarized by a function generator (Agilent 33220A, Santa Clara, CA) which applies an AC electric field of 5 MHZ and 20 $V_{p.p.}$ for 2 minutes, creating a force known as dielectrophoresis to attract the cells to the edges of the microtip. The microtip with captured cells is quickly rinsed in SDS solution (0.1% in DI water) to remove non-specific cells that are not bound to immobilized antibodies (Fig. 2-2). The tip is then translocated to the fluorescent antibody solution (10 μL, 2 mg/mL) where the remaining cells are labeled (Fig. 2-3). The microtip is lastly rinsed in DI water (160 μL) to remove unbound fluorescent antibodies (Fig. 2-4). The fluorescence images of microtips (100X

total magnification) are captured under a fluorescence microscope (Olympus BX-41, Olympus America Inc., Melville, NY). Fluorescent intensity is digitally measured and analyzed to yield a numeric result.

Although the microtip assay is now semi-automated, microtip production remains partially manual. As a result it requires quality control of the microtips to compensate for the reduced precision from manual functionalization. The quality of microtips was controlled by measuring the auto-fluorescence from the microtips after immobilization of antibodies using an excitation and emission filter of 495 nm and 520 nm, respectively. Auto-fluorescence is correlated to the thickness of the functionalized protein layers. Microtips with too high or low auto-fluorescence were discarded. By using this measurement to cull improperly functionalized microtips, the signal-to-noise ratio was improved at the specific range of the auto-fluorescence. This process, however, will be completed by the manufacturer and would not concern the clinic or hospital operating the assay.

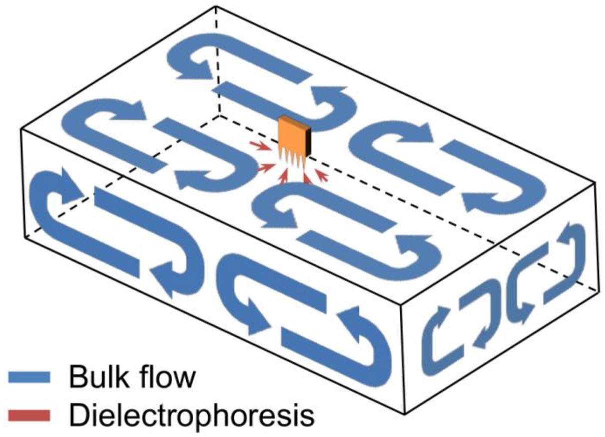

Figure S1 Rapid concentration mechanism using a combination of bulk fluid flow and dielectrophoresis.
